# Case report: Anti-neurexin-3α-associated autoimmune encephalitis secondary to contrast-induced encephalopathy

**DOI:** 10.3389/fneur.2023.1060110

**Published:** 2023-03-06

**Authors:** Lin Zhu, Qunzhu Shang, Charlie Weige Zhao, Shujuan Dai, Qian Wu

**Affiliations:** ^1^Department of Neurology, First Affiliated Hospital, Kunming Medical University, Kunming, Yunnan, China; ^2^Department of Internal Medicine, St. Vincent's Medical Center, Bridgeport, CT, United States

**Keywords:** anti-neurexin-3α-associated autoimmune encephalitis, contrast-induced encephalopathy (CIE), blood brain barrier, neuroimmunology, Q_Alb_ (CSF/serum albumin quotients)

## Abstract

A 54-year-old man complained of episodic stinging in his left eye along with weakness and numbness in his right upper and lower extremities for 1 month. The neurological examination was negative. MRI showed bilateral paraventricular demyelination. CTA showed significant stenosis of the left internal carotid (60%) and vertebral arteries (70%). He underwent left internal carotid stenting and was intubated during the procedure. After the procedure, he did not wake up from anesthesia, and he developed flexion and spasticity in the right arm immediately. Thereafter, he was sent to the neurocritical unit (NCU). Anti-seizure treatment was adopted due to recurrent general tonic-clonic seizures. Two days later (day 15 of hospitalization), brain edema and meningitis appeared in MRI, and contrast-induced encephalopathy (CIE) was mainly considered, with the support of CSF results. After 18 days (day 21 of hospitalization), serum anti-neurexin-3α IgG was detected at a dilution of 1:10. Anti-neurexin-3α-associated encephalitis was diagnosed. The patient was fully recovered 7 months after taking immunoglobulin, steroids, mortimycophenate, and cyclophosphamide. Meanwhile, anti-neurexin-3α antibody IgG was negative in both CSF and serum. MRI was also normal. Although scarce evidence clarified the relationship between CIE and anti-neurexin-3α-associated encephalitis, we inferred that the BBB damaged by CIE may result in the anti-neurexin-3α antibody entrance into the CSF from serum, which led to autoimmune encephalitis (AIE).

## Introduction

Contrast-induced encephalopathy (CIE) is a transient and reversible adverse reaction of the nervous system to the use of contrast, characterized by seizures, cortical blindness, global aphasia, focal neurological deficits, and chemical meningitis (1). The risk factors of CIE include large contrast volume (80–400 mL), chronic hypertension, transient ischemic attacks (TIAs), impaired cerebral autoregulation, impaired renal function, vertebral-basilar arteriography, male gender, and previous adverse reactions to contrast (2–4). In 2016, Gresa et al. first reported five patients diagnosed with anti-neurexin-3α-associated autoimmune encephalitis (AIE) (5). Few cases of anti-neurexin-3α-associated AIE have been reported since then. Herein, we report a patient who was diagnosed and subsequently recovered from anti-neurexin-3α-associated AIE secondary to CIE.

## Case report

A 54-year-old man with no significant history complained of episodic stinging pain in his left eye along with weakness and numbness in his right upper and lower extremities for 1 month. The episodes initially lasted for 3–5 min each and occurred only during the daytime but progressively increased in frequency and duration to last up to 10–15 min and occurred during the night. The patient had not gone to the hospital until he developed progressively worsening symptoms. His neurological examination was unremarkable, and the brain MRI showed bilateral paraventricular demyelination.

**Figure 1:**
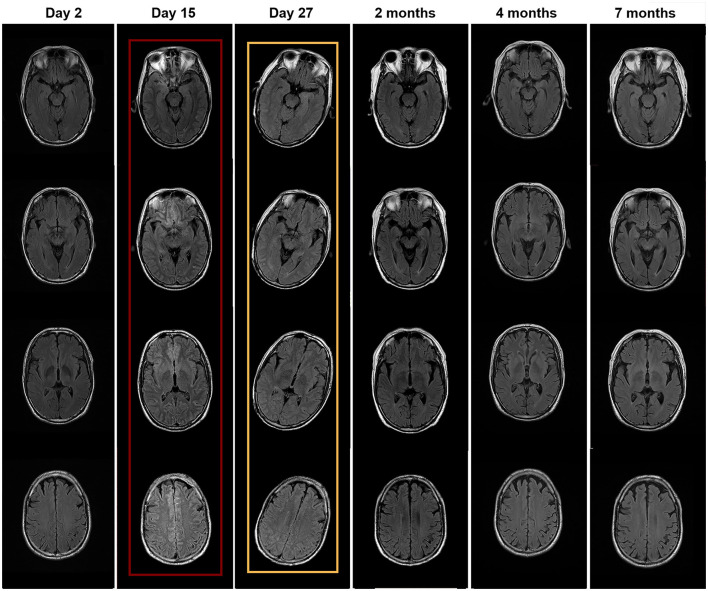
Brain MRI performed at different times. MRI with red wireframe was performed 2 days after surgery (day 15 of hospitalization) and showed brain edema and high signal in the meninges. MRI with yellow wireframe was performed on day 27 and showed improvement in abnormal findings.

The episodes were suggestive of TIAs and the patient underwent computed tomography (CT) angiography, which showed significant stenosis of the left internal carotid (60%) and vertebral (70%) arteries. He underwent left internal carotid artery stenting following 200 ml of intravenous contrast administration and was intubated during the procedure. After the procedure, he did not regain consciousness, and half an hour later, he developed flexion and spasticity in the right arm. His temperature was 36.5°C, heart rate was 110/min, blood pressure was 148/106 mmHg, respiratory rate was 22/min, and SpO_2_ was 98% (on ventilator support). The pupillary diameter was 1.5 mm in ambient light, and the pupillary light reflex was sluggish. On motor examination, he had hypertonia in the extremities and trunk and showed abnormal flexion to pain. His Glasgow Coma Scale score was 4T (E1VTM3). The patient developed recurrent generalized tonic-clonic seizures, lasting from 10 s to several minutes. The lactic acid in his blood was high (12.3 mmol/L). He was treated with midazolam and vecuronium. The Richmond Agitation Sedation Scale score was −4. The control of seizures required continuous intravenous infusion of midazolam for 48 h. Subsequently, oral levetiracetam was administered to allow a reduction in the midazolam dose. Several investigations were performed 2 days later (day 15 of hospitalization): His brain MRI showed edema and generalized high signal in the meninges (Figure 1). His electroencephalogram showed low voltage with no epileptiform discharges. The abdomen and testicle ultrasound were normal; the CT chest was normal. Rheumatism immune-related antibodies, including antinuclear antibody (ANA), antineutrophil cytoplasmic antibody (ANCA), anti-ribonucleoprotein antibody (anti-RNP), double-stranded DNA (anti-dsDNA), and Sjögren syndrome-related antigens A and B (anti-SS-A/SS-B), were negative. In addition, the T-cell-based test for tuberculosis infection and the tuberculin skin test were negative. The result of the cerebrospinal fluid (CSF) analysis is presented in Table 1. The patient was empirically treated with the antiviral medication acyclovir.

**Table 1:** Laboratory data.

**Laboratory data**							
		**Day 17**	**Day 21**	**Day 29**	**2 months**	**4 months**	**7 months**
CSF	Pressure (mmH_2_0)	230	175	190	155	205	190
	WBC (10^6^/L)	67	12	4	1	6	1
	RBC (10^6^/L)	12	2	12	0	2	0
	Cl^−^ (mmol/L)	138.8	130.2	131.1	129.2	129.0	136.8
	Glu (mmol/L)	6.32	5.74	3.54	3.05	3.41	4.57
	Album (mg/L)	442.3	233.4	134.2	248.2	285.8	192.7
	Pro (g/L)	0.47	0.24	0.61	0.96	1	0.24
	Anti-neurexin-3α antibody IgG	/	-	-	1:10	-	-
Blood	Anti-neurexin-3α antibody IgG	/	1:10	-	1:32	1:10	-
	Album (mg/L)	36.5	37	28.5	40.2	36.7	38.4
	Glu (mmol/L)	8.9	7.5	6.8	6.5	/	/
	Glu (CSF/Blood)	0.71	0.76	0.52	0.47	/	/
	Q_Alb_ (‰)	12.12	6.31	4.71	6.17	7.79	5.01

After 21 days of his hospitalization, serum anti-neurexin-3α IgG came back positive with a ratio of 1:10. The anti-neurexin-3α IgG antibodies were not detected in the CSF. The patient was diagnosed with anti-neurexin-3α-associated AIE and treated with a single dose of IV immunoglobulin (0.4 g/kg/day for 5 days). In the following days, the patient developed psychiatric symptoms, including agitation, auditory and visual hallucinations, cognitive dysfunction, and significantly reduced sleep duration. His Bech–Rafaelsen Mania Rating Scale score was 23, indicating severe mania. He was treated with olanzapine to control psychiatric symptoms. On day 28 of hospitalization, CSF analysis was repeated, which was normal, except for raised proteins, and the anti-neurexin-3α antibody was negative in both serum and CSF (Table 1). The brain MRI showed improved both brain edema and high signal in the meninges (Figure 1). Steroids were added to the patient's treatment.

**Figure 2:**
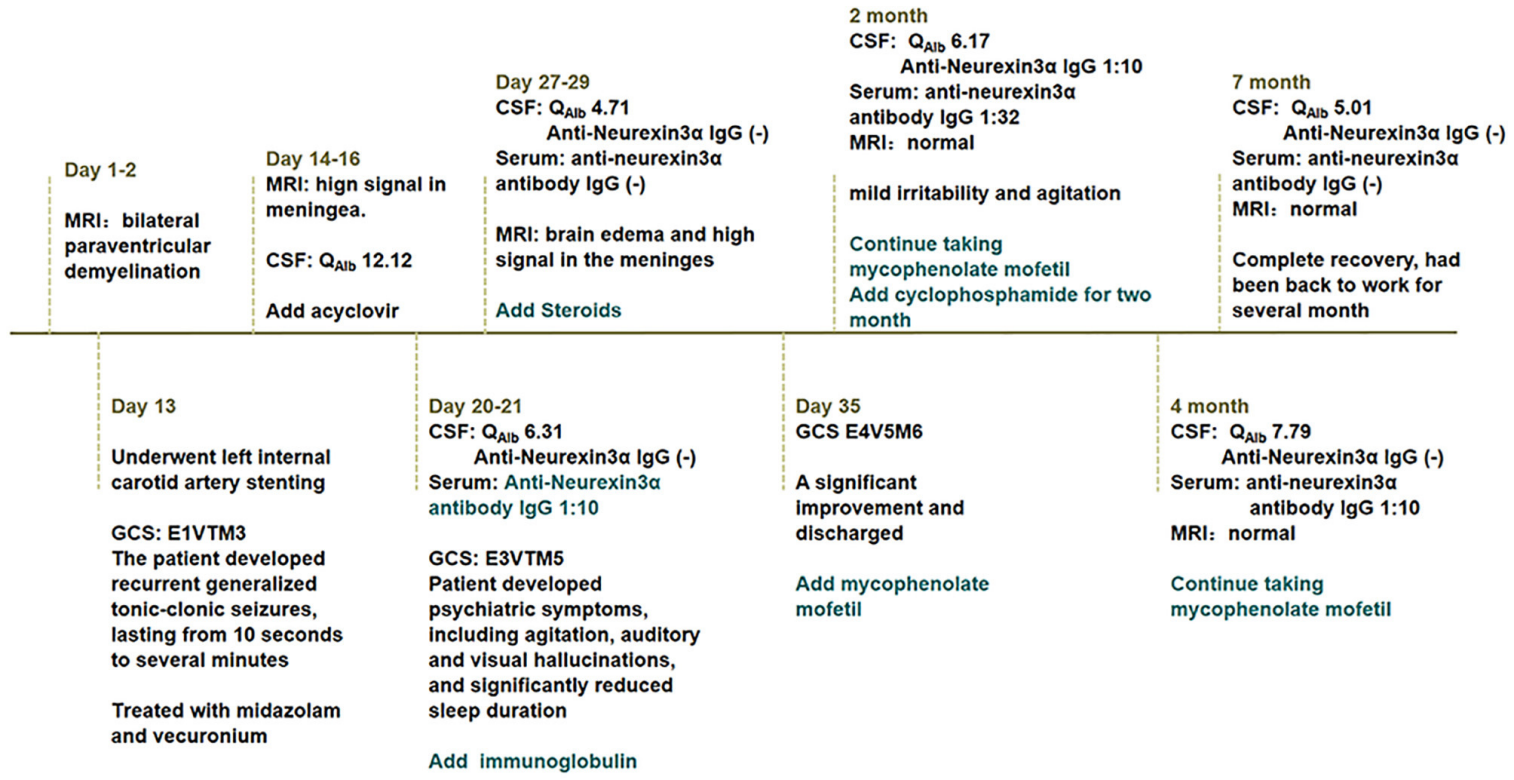
Case timeline.

On day 35 of hospitalization, the patient was discharged because of significant improvement and was asked to continue mycophenolate mofetil. The patient presented for follow-up after 2 months and reported significant improvement in the psychotic symptoms, with only mild irritability and agitation. His CSF Q_Alb_ was 6.17, anti-neurexin3α IgG was positive at 1:10, and serum anti-neurexin-3α antibody IgG was 1:32. Therefore, we added cyclophosphamide for 2 months in addition to mycophenolate mofetil. After 4 months, his CSF Q_Alb_ was 7.79, anti-neurexin-3α antibody IgG was negative, serum anti-neurexin-3α antibody IgG was 1:10, and the brain MRI was normal. Thus, the patient was asked to continue mycophenolate mofetil. After 7 months, both the CSF analysis and the brain MRI were normal (Figure 1), the anti-neurexin-3α antibody IgG was negative in CSF and serum, and the patient had a complete recovery and had been back to work for several months. The case timeline was showed in Figure 2.

## Discussion

**Figure 3:**
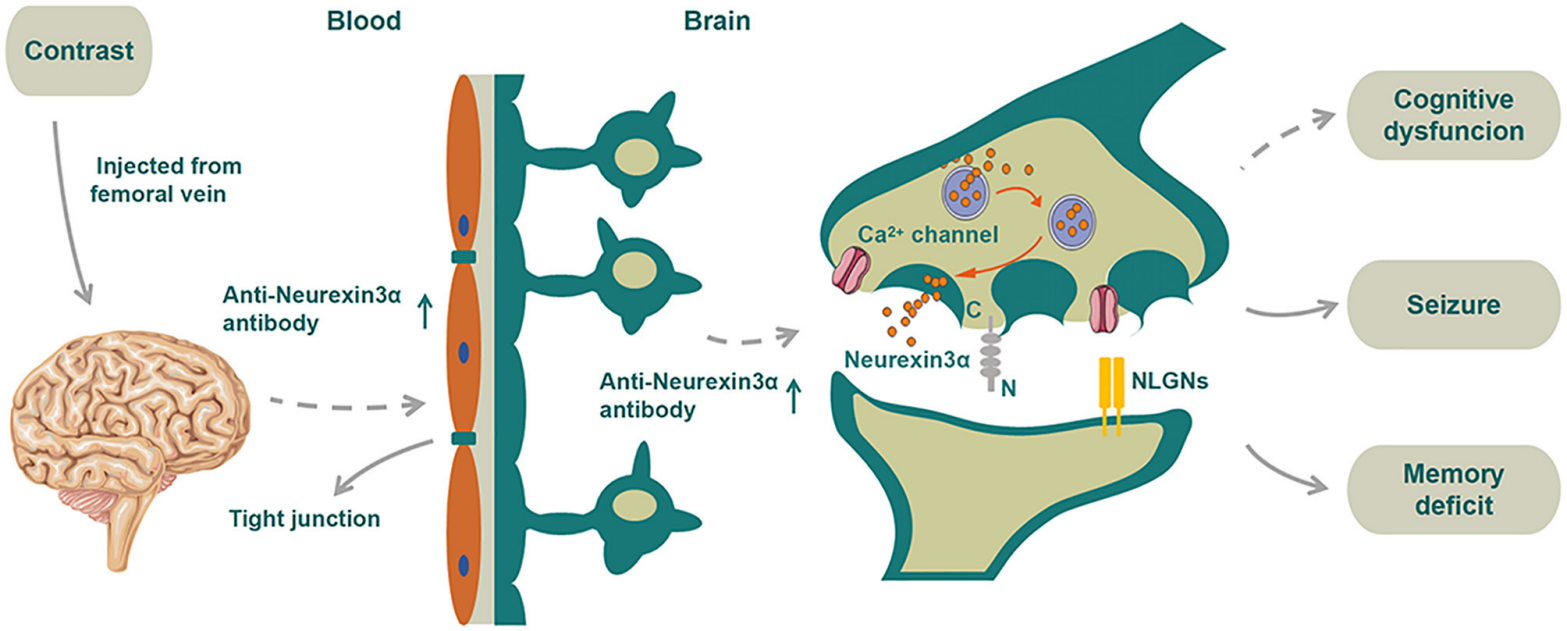
Schematic diagram of the proposed mechanism of disease for the patient. We speculated that neurexin-3α may be related to T-Js and the neurexin-3α antibody may promote BBB damage. As a vicious circle, the damaged BBB may allow more entrance of neurexin-3α antibodies to the CSF from serum. Contrast agents that destroyed the BBB would further assist manyfold neurexin-3α antibodies to enter the CSF, thereby disabling the function of neurexin-3α in the presynaptic membrane, impairing the activity of calcium ion channels and eventually leading to a series of neurological symptoms.

The major clinical manifestations in our patient were first considered as TIA-like symptoms in the emergency department. He had critical stenosis of the internal carotid artery, for which it was reasonable to perform endovascular treatment. The patient received cerebral angiography due to repeated presentation of TIA-like symptoms although his left internal carotid stenosis was only around 60%. After the surgery, the patient progressed to develop coma, myoclonic jerks, and psychotic symptoms after the surgery. His brain MRI showed high signals in the meninges. The Q_Alb_ [CSF/serum albumin quotients, which is a marker of the blood–brain barrier function with the upper reference limit 8 × 10^−3^ for patients aged <60 years (6)] was increased (Table 1), suggesting BBB damage. Initially, we speculated that the BBB was damaged due to the contrast, which increased the BBB permeability, resulting in coma and myoclonic jerks; therefore, the diagnosis of contrast-induced encephalopathy (CIE) was considered. However, 20 days later, anti-neurexin-3α antibody IgG was detected in serum, and the patient developed extreme agitation, auditory and visual hallucinations, and significantly reduced sleep duration; thus, the patient was diagnosed with anti-neurexin-3α-associated AIE for which he received immunotherapy. The repeat CSF test also showed positive anti-neurexin-3α antibody IgG. The antibody entered the brain from the blood due to the damaged BBB, which impaired the activity of calcium ion channels and led to several neurological symptoms, including agitation, auditory and visual hallucinations, and significantly reduced sleep duration. To sum up, we inferred that the CIE may have led to BBB damage, which allowed the antibodies to enter the CSF from the serum, eventually leading to anti-neurexin-3α-associated AIE (Figure 3).

At present, there are no clear diagnostic criteria for CIE. Chu et al. analyzed a group of patients with CIE and proposed that CIE should be considered if symptoms worsen after endovascular thrombectomy and brain imaging suggest edema and contrast agent residue, especially in patients with renal dysfunction or previous stroke (7). Similarly, Monforte et al. reported a CIE patient with previous chronic renal dysfunction who developed altered consciousness and myoclonic jerks of the right arm after left internal carotid stenting for critical stenosis (8). To sum up, TIA and endovascular treatment were the risk factors of CIE in the patient. Based on the clinical and MRI findings, we diagnosed CIE.

Currently, the specific mechanism underlying brain injury caused by contrast agents is unclear. Some researchers have proposed that the chemical toxicity of contrast agents can cause the contraction of endothelial cells, destroy the close connection between them, and promote endocytosis and extremes of endothelial cells (9). Wang et al. found that a carotid injection of iohexol damages the BBB in rats and caused dynamic variability in BBB permeability, with progressively increasing permeability, reaching a maximum at 6 h after administration, and then progressively decreasing. The underlying mechanism may be related to the changes in the expression of tight junction (T–J)-related proteins (ZO-1 and occludin) in the BBB (10).

Interestingly, the anti-neurexin-3α antibody was initially detected in the patient's serum and then in the CSF. Neurexins are cell-surface glycoproteins encoded by NRX. Neurexin-3α is the largest and most polymorphic member of the neurexin family. Its C-terminus is involved in the regulation of Ca^2+^ channel function and neurotransmitter release, whereas the N-terminus, which is outside the presynaptic membrane binds to the adhesion protein in the postsynaptic membrane (11, 12) to regulate the formation and function of synapses. In neurexin-α knock-out mice, the lack of function of the presynaptic Ca^2+^ channel (11) leads to severely reduced neurotransmitter release. Some scholars have proposed that the neurexin and neuroligin complex may be related to cognitive diseases, but the underlying mechanism remains to be studied (13).

In 1996, Baumgartner et al. reported that neurexin IV was a new member of the neurexin family located at the septate junctions (S-Js) between the epithelial cells and glial cells; the loss of neurexin IV in S-Js led to BBB destruction (14). The T-Js in vertebrates correspond to the S-Js in invertebrates; thus, we speculated that neurexin-3α may be related to T-Js and the neurexin-3α antibody may promote BBB damage. As a vicious circle, the damaged BBB may allow more entrance of neurexin-3α antibodies to the CSF from serum, thereby disabling the function of neurexin-3α in the presynaptic membrane, impairing the activity of calcium ion channels, and eventually leading to a series of neurological symptoms. In addition, whether the initial TIA-like symptoms might have been focal seizures that induced by AIE were impossible to verify due to absence of immediate EEG and preoperative antibody detection, but this diagnosis should also be taken into consideration. If this assumption was right, contrast agents that destroyed the BBB would further assist manyfold neurexin-3α antibodies to enter the CSF, thereby disabling the function of neurexin-3α in the presynaptic membrane, impairing the activity of calcium ion channels and eventually leading to a series of neurological symptoms (Figure 3).

In conclusion, we should be vigilant against CIE in patients who have a transient and reversible adverse reaction of the nervous system after the use of contrast. In addition, AIE may be secondary to or complicated by a neurological disease, especially in patients with immunosuppression. AIE should be considered when focal neurological episodes presented a comprehensive work-up of MRI; EEG and autoimmune antibody detection are helpful for diagnosis and management. Moreover, this rare case deserves attention and can provide a reference for clinicians.

## Data availability statement

The original contributions presented in the study are included in the article/supplementary material, further inquiries can be directed to the corresponding authors.

## Ethics statement

Written informed consent was obtained from the individual(s) for the publication of any potentially identifiable images or data included in this article.

## Author contributions

LZ drafted the manuscript. QS managed the patient throughout and participated in the discussion of writing the article to provide ideas. CZ provided ideas for writing and language modification. QW and SD guided patient treatment and article writing ideas, and these authors contributed equally to this study and share corresponding authorship. All authors contributed to the article and approved the submitted version.
